# Resolution of bone, cutaneous, and muscular involvement after haploidentical hematopoietic stem cell transplantation followed by post‐transplant cyclophosphamide in adult T‐cell leukemia/lymphoma

**DOI:** 10.1002/ccr3.2925

**Published:** 2020-06-02

**Authors:** Futoshi Iioka, Hiroshi Tanabe, Gen Honjo, Takashi Misaki, Hitoshi Ohno

**Affiliations:** ^1^ Departments of Hematology Tenri Hospital Tenri Japan; ^2^ Departments of Dermatology Tenri Hospital Tenri Japan; ^3^ Departments of Diagnostic Surgical Pathology Tenri Hospital Tenri Japan; ^4^ Departments of Radioisotope Center Tenri Hospital Tenri Japan

**Keywords:** ^18^F‐FDG‐PET, adult T‐cell leukemia, CT, haploidentical hematopoietic stem cell transplantation, lymphoma, muscular involvement, post‐transplant cyclophosphamide

## Abstract

Haploidentical hematopoietic stem cell transplantation followed by post‐transplant cyclophosphamide provides a well‐tolerated and potentially curable treatment for chemorefractory acute‐type adult T‐cell leukemia/lymphoma.

## Graphical abstract

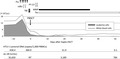

## 1 INTRODUCTION

We herein present a female patient with acute‐type adult T‐cell leukemia/lymphoma that progressed from the chronic type. Her disease involved bone, skin, and skeletal muscles predominantly in the lower legs. She underwent haploidentical hematopoietic stem cell transplantation, leading to the resolution of extralymphatic involvement within 2 years.

Adult T‐cell leukemia/lymphoma (ATLL) is listed as a category of mature T‐ and NK‐cell neoplasms in the 2017 WHO classification scheme of hematolymphoid tumors.^1^ ATLL is unique in that the tumor is etiologically linked to human T‐cell lymphotropic virus type 1 (HTLV‐1), which is endemic in certain areas of Japan. Since patients with ATLL present with diverse manifestations, the disease has been classified into smoldering, chronic, lymphoma, and acute subtypes,^1^, ^2^ and progression from indolent to aggressive subtypes may occur. The most common form is the acute subtype, which is characterized by florid leukemia picture with bone marrow (BM) infiltration, generalized lymphadenopathy, hepatosplenomegaly, and hypercalcemia with or without lytic bone lesions. Cutaneous lesions are found in >50% of cases.^1^

The treatment of ATLL has been challenging because patients generally do not respond or have only transient responses to chemotherapy regimens, and high‐dose chemotherapy with autologous hematopoietic stem cell transplantation (HSCT) is not recommended due to the high rates of relapse and HSCT‐related mortality.^2^ Allogeneic HSCT (allo‐HSCT) is the only potentially curative treatment currently available for patients with ATLL.^3^, ^4^

We encountered a female patient with ATLL whose disease involved bone, skin, and skeletal muscle. She underwent allo‐HSCT from a human leukocyte antigen (HLA)‐haploidentical son and showed a favorable post‐transplant course. We herein describe her clinical features, imaging studies, and treatment course in detail.

## 2 CASE PRESENTATION

A 49‐year‐old woman was referred to the hematology department because an examination of peripheral blood smear slides revealed neoplastic lymphocytes; her white cell count was 9.2 × 10^3^/µL with 49% lymphocytes (absolute number, 4.5 × 10^3^/µL), including neoplastic cells with an irregular nuclear morphology. Flow cytometry showed that these cells were CD2^+^, CD3^+^, CD4^+^, CD5^+^, CD7^−/dim^, CD8^−^, CD25^+^, CD122^−^, and CCR4^+^. An examination of a BM aspirate smear revealed 18.7% neoplastic lymphocytes, and biopsy showed interstitial infiltrates of CD3^+^ neoplastic cells. She was seropositive for HTLV‐1. Lactate dehydrogenase (LDH), serum albumin, urea nitrogen, and serum calcium levels were within normal ranges. No lymphadenopathy or hepatosplenomegaly was found. Her disease was then classified into the chronic type of ATLL, and a watchful waiting policy was adopted. She underwent breast‐conserving surgery for breast cancer 7 years earlier and did not receive adjuvant chemotherapy or radiotherapy.

**FIGURE 1:**
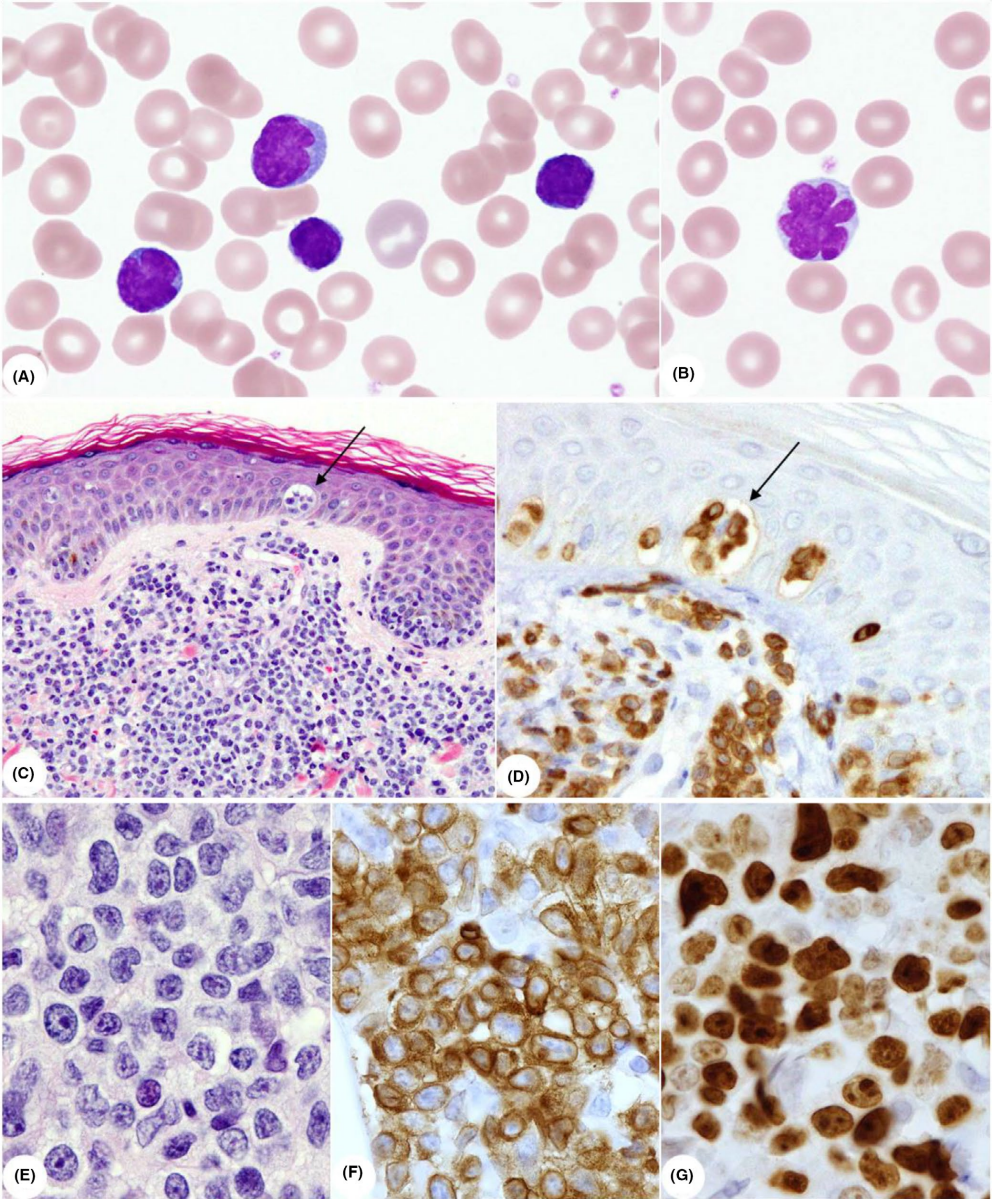
Images of peripheral blood and skin biopsy. *Top*. Wright‐stained leukemia cells. A, Small to medium‐sized leukemia cells with irregular nuclei and condensed chromatin and B, a “flower cell.” Original magnification, ×100 objective lens. *Middle*. Low‐power microscopic images of the skin. C, Hematoxylin and eosin (H&E) staining (×10) and D, anti‐CD3 immunostaining (×20). Arrows indicate Pautrier‐like microabscesses. *Bottom*. High‐power microscopic images of the skin, showing the appearance of neoplastic cells in the dermis. E, H&E staining (×100); F, anti‐CD3 immunostaining (×100); and G, anti–Ki‐67 immunostaining (×100)

**FIGURE 2:**
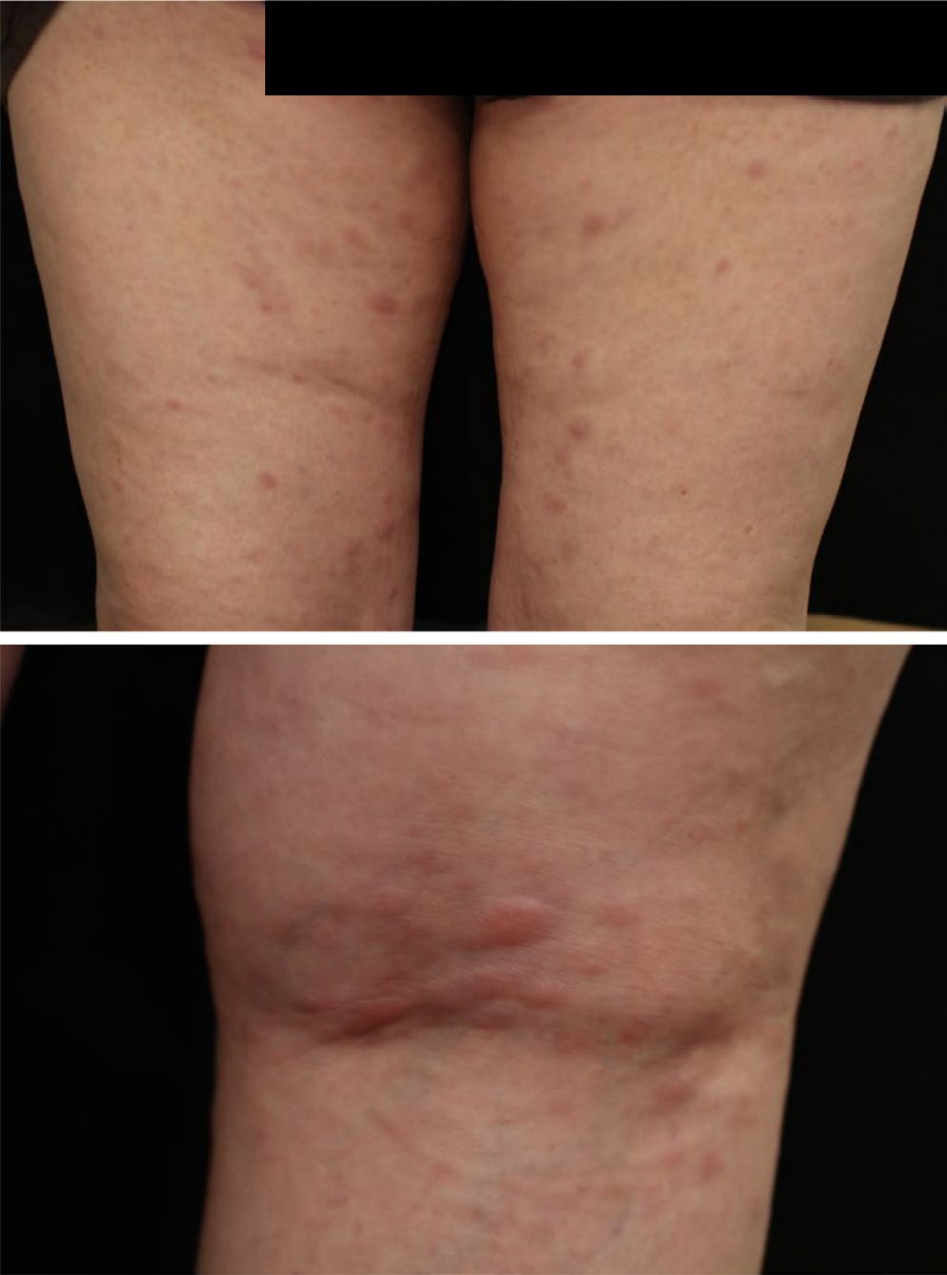
Images of thighs (*top*) and the popliteal fossa (*bottom*), showing multiple erythematous nodules

**FIGURE 3:**
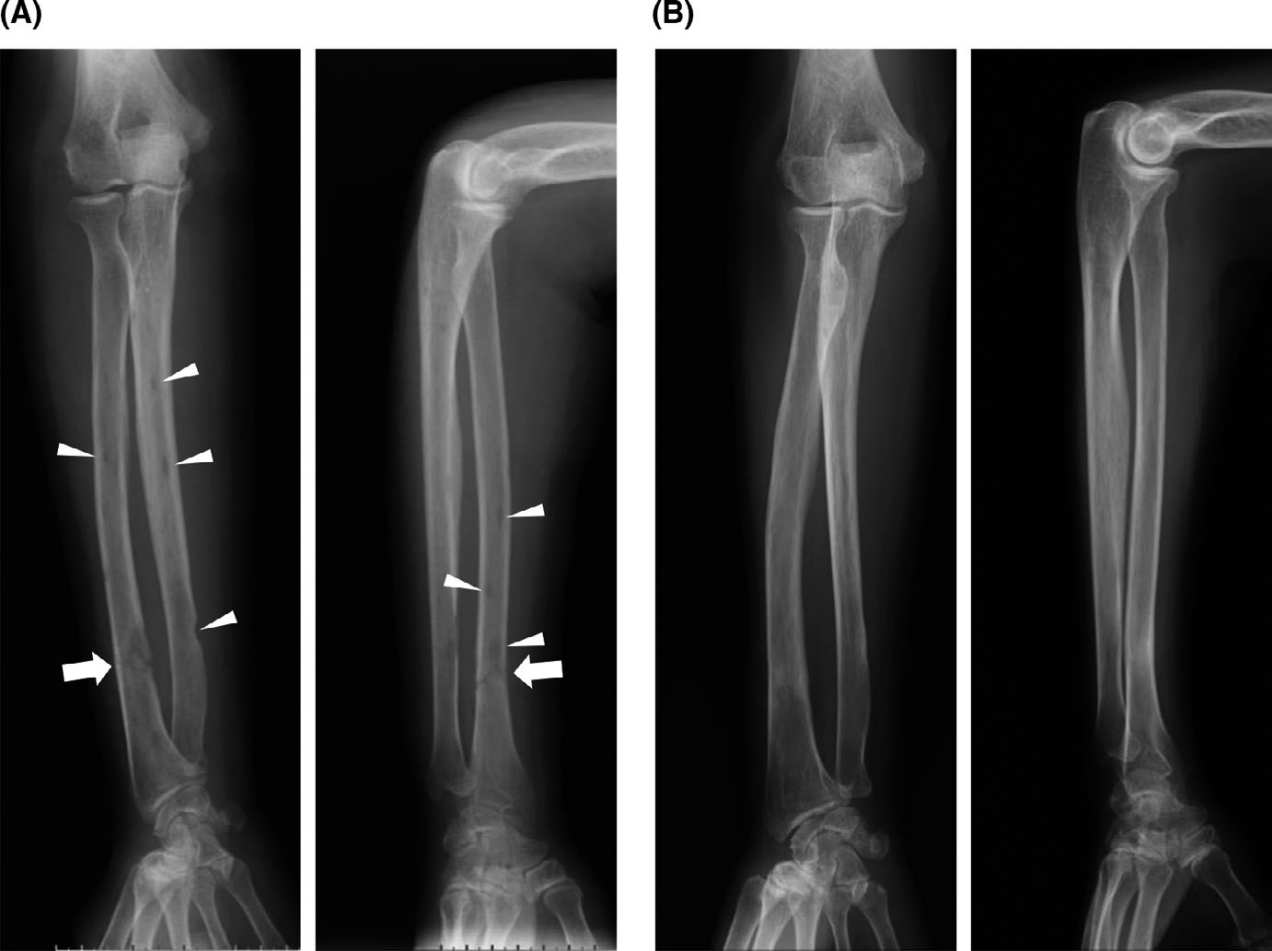
Plain radiographs of right forearm bones obtained before the initiation of chemotherapy (A) and 2 y after haplo‐HSCT (B). Arrows indicate the site of fracture of the radius. Osteolytic lesions are indicated by arrowheads

Six years after the initial presentation, her disease progressed to acute‐type ATLL, showing a marked increase in leukemia cells with multilobed nuclei and a basophilic cytoplasm (Figure 1, *top*) as well as elevated levels of LDH. She developed multiple erythematous lumps associated with subcutaneous infiltration in the lower extremities, which were detected by palpation (Figure 2). A pathological fracture of the distal corpus of the right radius occurred when she opened the lid of a bottle, and multiple lytic bone lesions were detected in the corpus of the radial and ulnar bones (Figure 3A). At the age of 56 years, her hemoglobin level was 11.9 g/dL, white cell count 36.46 × 10^3^/µL with 78.0% leukemia cells, and platelet count 316 × 10^3^/µL. Her urea nitrogen level was 12.3 mg/dL, creatinine 0.4 mg/dL, uric acid 5.3 mg/dL, albumin 3.7 g/dL, LDH 457 U/L (reference range, 124‐222 U/mL), creatine kinase (CK) 21 U/L, calcium 9.2 mg/dL, C‐reactive protein 0.24 mg/dL, and soluble interleukin‐2 receptor (sIL‐2R) 40, 290 U/mL (reference range, 145 to 519 U/mL). HTLV‐1 proviral DNA was 445.7 copies per 1 × 10^3^ peripheral blood mononuclear cells.

## 3 DIFFERENTIAL DIAGNOSIS AND INVESTIGATIONS

**FIGURE 4:**
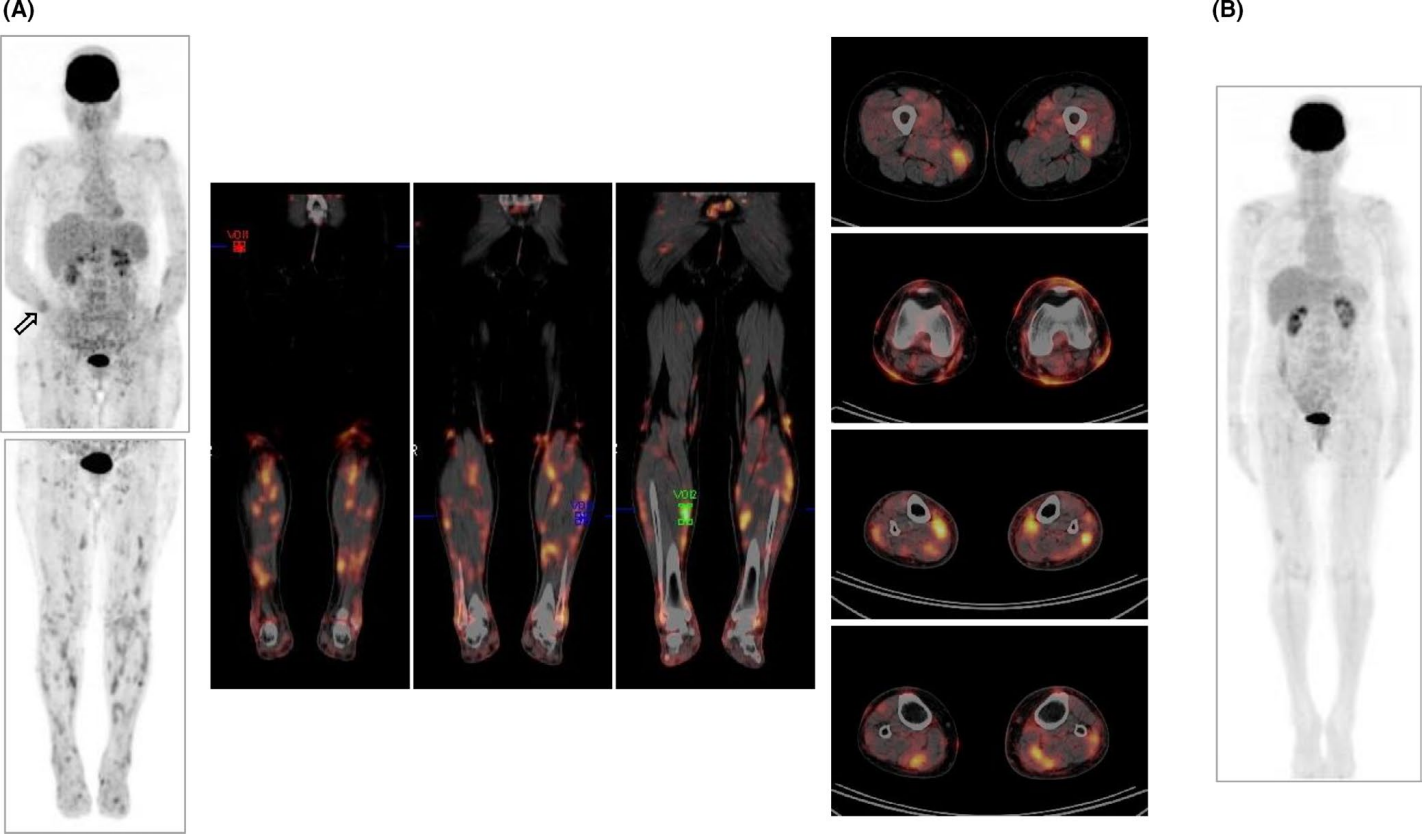
^18^F‐FDG‐PET/CT. (A) Images obtained before the initiation of chemotherapy. Anterior view of maximum intensity projection (MIP) images of the body (*left*) and representative coronal (*middle*) and axial (*right*) PET/CT fusion images of the legs are shown. The site of the radial fracture is indicated by an arrow. The maximal standardized uptake value of the muscular lesions was 5.16. (B) Anterior view of an MIP image of the body obtained on day 49 of haplo‐HSCT

Positron emission tomography (PET) combined with computed tomography (CT) revealed the slightly increased uptake of ^18^F‐fluorodeoxyglucose (FDG) throughout the BM space and in the enlarged spleen, and tracer‐avid lymph nodes were found in the subclavicular, hepatic hilar, and inguinal regions (Figure 4A). The tracer accumulated not only in the skin, in accordance with the cutaneous lumps identified by the physical examination, but also in skeletal muscles predominantly in both lower legs. Skin lesions appeared to extend to the subcutaneous tissues, but had no connection with the underlying muscular lesions. Bone lesions were not apparent on ^18^F‐FDG‐PET/CT, except for the site of the radial bone fracture (Figure 4A).

Biopsy of cutaneous lesions in the right popliteal fossa revealed the diffuse infiltration of large neoplastic cells extending from the dermis to the subcutaneous fat layer as well as Pautrier‐like microabscesses in the epidermis (Figure 1, *middle* and *bottom*). Immunohistochemistry showed that these cells were CD3^+^, CD5^+^, CD7^−^, CD20^−^, CD79a^−^, CD30^−^, CD20^−^, CD79a^−^, and ALK^−^. Ki‐67 positivity was more than 90% in the majority of proliferative areas (Figure 1, *bottom*).

## 4 TREATMENT AND OUTCOME

Her disease was refractory to the multiagent modified LSG15 protocol (VCAP‐AMP‐VECP: vincristine, cyclophosphamide, doxorubicin, and prednisolone; doxorubicin, ranimustine, and prednisolone; and vindesine, etoposide, carboplatin, and prednisolone) and DeVIC (dexamethasone, etoposide, ifosfamide, and carboplatin) salvage treatment. She was intolerant of lenalidomide due to a toxic skin rash. Fludarabine was ineffective. The patient then underwent allo‐HSCT from her son, who was HLA‐haploidentical and seronegative for HTLV‐1. The conditioning regimen consisted of fludarabine (30 mg/m^2^ daily on days −6 to −2), intravenous busulfan (3.2 mg/kg daily on days −4 and −3), and total body irradiation (2 Gy per fraction, twice on day −1).^5^ On day 0, granulocyte colony‐stimulating factor‐mobilized blood stem cells of 7.3 × 10^6^ CD34^+^ cells per kilogram were transfused. Acute graft‐vs‐host disease (GVHD) was prevented by post‐transplant cyclophosphamide (PT‐CY, 50 mg/kg daily on days 3 and 4), tacrolimus, and mycophenolate mofetil.^5^ Neutrophil engraftment was achieved on day 15, and BM obtained on day 36 revealed normal hematopoietic precursors with no residual leukemia cells. Sex chromosome‐specific interphase fluorescence in situ hybridization confirmed complete donor chimerism. She developed grade 1 acute GVHD with stage 2 cutaneous involvement on day 77, which was resolved by low‐dose prednisolone. Tacrolimus was appropriately tapered and withdrawn 14 months after the transplant. No significant infectious complications occurred.

**FIGURE 5:**
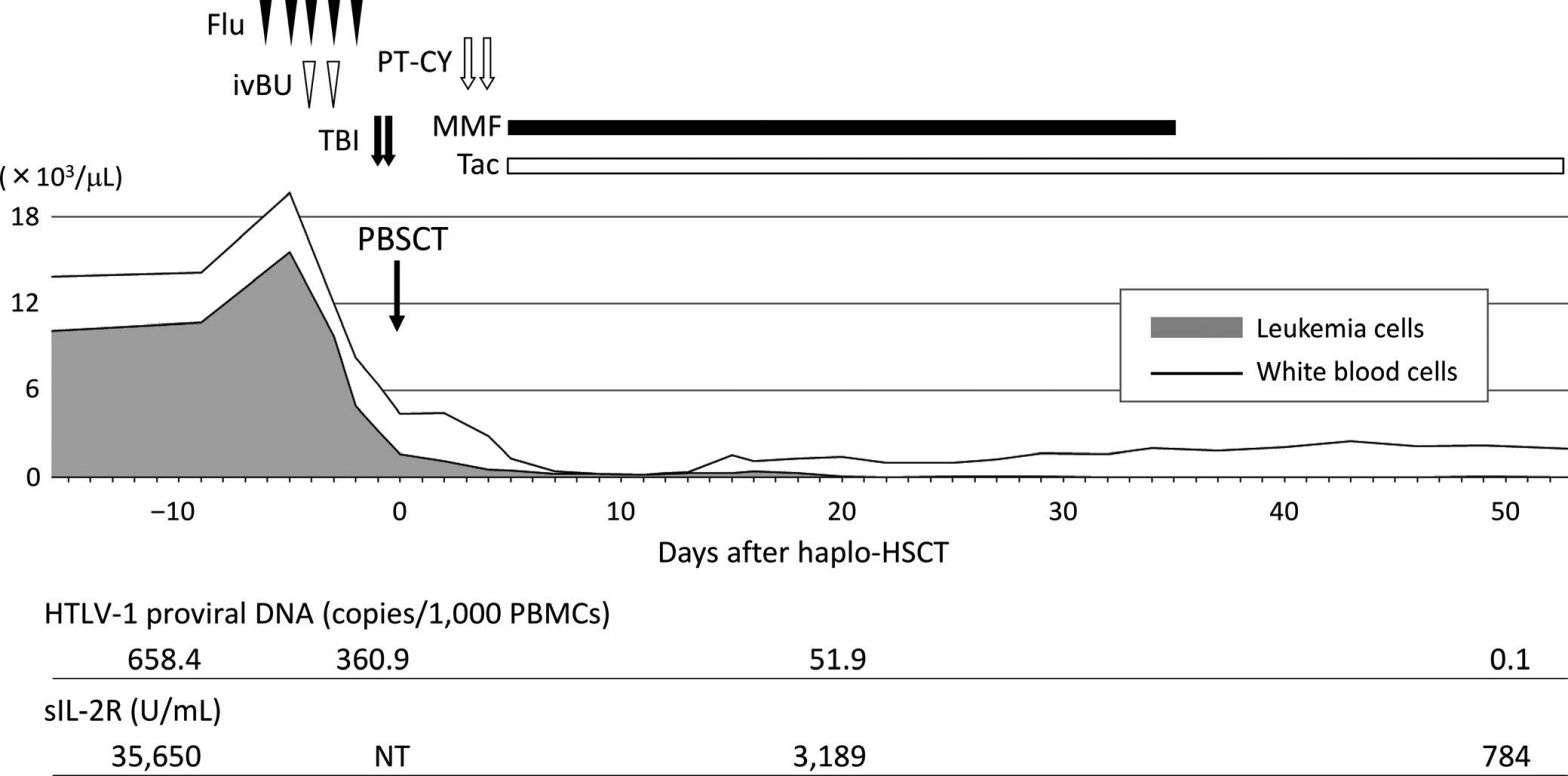
The course of haplo‐HSCT followed by PT‐CY. Details of the schedule, dosages of the conditioning regimen, and prophylaxis for acute GVHD are described in the text. Counts of white blood cells and leukemia cells in peripheral blood are shown. The values of HTLV‐1 proviral DNA and sIL‐2R on days − 10, 0, 20, and 50 are shown in the *bottom*. Flu, fludarabine; ivBU, intravenous busulfan; TBI, total body irradiation; PBSCT, peripheral blood stem cell transplantation; PT‐CY, post‐transplant cyclophosphamide; MMF, mycophenolate mofetil; Tac, tacrolimus; and NT, not tested

In response to the conditioning regimen, leukemia cells were readily eradicated from the blood and regression of the tumor burden was supported by the favorable courses of sIL‐2R values and copies of HTLV‐1 proviral DNA after the transplant (Figure 5). ^18^F‐FDG‐PET/CT on day 49 showed the resolution of cutaneous and muscular lesions and the avidities of BM and the spleen became normalized (Figure 4B). Osteolytic lesions in the right forearm bones were confirmed to be resolved by radiography (Figure 3B). She has remained free from progression for 2 years after the transplant.

## 5 DISCUSSION

We herein described the course of a woman with chemorefractory ATLL who presented with unusual extralymphatic involvement. She favorably responded to haplo‐HSCT followed by PT‐CY and has been free from progression for more than 2 years, suggesting that this approach provides a well‐tolerated and potentially curable treatment for this difficult‐to‐treat disease.

^18^F‐FDG‐PET/CT is effective for initial staging and response evaluations in Hodgkin's lymphoma and diffuse large B‐cell lymphoma.^6^ However, the role of this imaging modality has not yet been fully defined in ATLL. In a study on 135 patients with T‐cell lymphoma, including nine with ATLL, 122 (90%) had FDG‐avid diseases and 39 (29%) had sites of disease beyond the range of a diagnostic neck, chest, abdomen, and pelvis CT scan.^7^ In our patient, we initially aimed to evaluate nodal and cutaneous diseases by ^18^F‐FDG‐PET/CT, but unexpectedly found evidence of muscular involvement. Thus, ^18^F‐FDG‐PET/CT detects involved organs or tissues that are invisible in a physical examination or CT. In contrast, it is important to note that lytic bone lesions recognized by plain radiographs were not detectable by ^18^F‐FDG‐PET/CT.

The involvement of skeletal muscles in ATLL is unusual. A literature review found a single case report of a female patient whose disease involved almost all muscles in the body and biopsy showed the destructive infiltration of neoplastic cells expressing the p40Tax viral protein into the muscular tissues, leading to an elevated level of serum CK.^8^ In contrast, the present case showed the predominant involvement of the lower leg muscles in association with cutaneous involvement, while enlargement of the fascicles of FDG‐avid muscles was not apparent and CK levels were not elevated throughout the course, suggesting that muscular tissues themselves may not have been affected; the patient did not have weakness in the legs. Nevertheless, the anatomical basis for these characteristic sites of involvement and the histopathology of involved muscular tissues remain unclear.

A Japanese nationwide retrospective study of 586 patients with ATLL who underwent allo‐HSCT between 1992 and 2009 showed a median overall survival (OS) of 9.9 months and a 3‐year OS rate of 36%.^9^ Survival was adversely associated with not being in disease remission and a performance status 2‐4 with estimated hazard ratios of 1.94 and 4.057, respectively. The cumulative incidence of treatment‐related mortality 1 year after transplant was 32.7% in patients who received myeloablative conditioning and 29.2% in those who received reduced‐intensity conditioning.^9^ Regarding donor stem cell sources, unrelated donor or unrelated cord blood was associated with inferior outcomes to those with HLA‐matched related donors.^9^, ^10^ However, since the availability of HLA‐matched sibling donors has decreased due to the aging of both patients and siblings,^3^ haplo‐identical donors within the immediate family have emerged as alternative stem cell sources.^11^ Allo‐HSCT from haploidentical donors (Haplo‐HSCT) has been revolutionized by the use of PT‐CY, targeting alloreactive T cells, thereby preventing acute GVHD.^3^, ^12^, ^13^

A significant concern regarding haplo‐HSCT followed by PT‐CY is that PT‐CY theoretically decreases the graft‐vs‐tumor effect, resulting in an increased risk of relapse.^11^ In the present case, a significant decrease in the HTLV‐1 proviral load and more than 2 years of progression‐free survival suggested that graft‐vs‐HTLV‐1 and graft‐vs‐tumor effects had been effectively functioning and these effects had contributed to the resolution of bone, cutaneous, and muscular involvement. In an ATLL patient who underwent haplo‐HSCT/PT‐CY, but relapsed early with skin involvement, the disease was resolved by the sequential use of mogamulizumab and lenalidomide with the aim of enhancing the immune response.^14^ Nevertheless, since information on haplo‐HSCT/PT‐CY in patients with ATLL is limited,^14^ the findings of a relevant phase II trial that is currently underway in Japan (UMIN000021783)^13^ are awaited in order to establish whether this approach may be safely and effectively performed for these patients.

## CONFLICT OF INTEREST

The authors have no conflicts of interest to disclose.

## AUTHOR CONTRIBUTIONS

All authors: have made substantial contributions to all of the following: FI: was the attending physician of the patient and obtained patient consent. HT: was involved in dermatology consulting. GH: performed the histopathological expert analysis. TM: performed the radiological expert analysis. FI and HO: were responsible for writing and reviewing the manuscript, the literature review, and overall revisions to the manuscript.

## CONSENT FOR PUBLICATION

Informed consent was obtained from the patient for the publication of this manuscript.
